# Pathway interactions between MAPKs, mTOR, PKA, and the glucocorticoid receptor in lymphoid cells

**DOI:** 10.1186/1475-2867-7-3

**Published:** 2007-03-28

**Authors:** Aaron L Miller, Anna S Garza, Betty H Johnson, E Brad Thompson

**Affiliations:** 1 Department of Biochemistry and Molecular Biology, University of Texas Medical Branch, 301 University Boulevard, Galveston, Texas 77555-1068, USA

## Abstract

**Background:**

Glucocorticoids are frequently used as a primary chemotherapeutic agent in many types of human lymphoid malignancies because they induce apoptosis through activation of the glucocorticoid receptor, with subsequent alteration of a complex network of cellular mechanisms. Despite clinical usage for over fifty years, the complete mechanism responsible for glucocorticoid-related apoptosis or resistance remains elusive. The mitogen-activated protein kinase pathway is a signal transduction network that influences a variety of cellular responses through phosphorylation of specific target substrates, including the glucocorticoid receptor. In this study we have evaluated the pharmaceutical scenarios which converge on the mitogen-activated protein kinase pathway to alter glucocorticoid sensitivity in clones of human acute lymphoblastic CEM cells sensitive and refractory to apoptosis in response to the synthetic glucocorticoid dexamethasone.

**Results:**

The glucocorticoid-resistant clone CEM-C1-15 displays a combination of high constitutive JNK activity and dexamethasone-induced ERK activity with a weak induction of p38 upon glucocorticoid treatment. The cells become sensitive to glucocorticoid-evoked apoptosis after: (1) inhibition of JNK and ERK activity, (2) stimulation of the cAMP/PKA pathway with forskolin, or (3) inhibition of mTOR with rapamycin. Treatments 1–3 in combination with dexamethasone alter the intracellular balance of phospho-MAPKs by lowering JNK phosphorylation and increasing the level of glucocorticoid receptor phosphorylated at serine 211, a modification known to enhance receptor activity.

**Conclusion:**

Our data support the hypothesis that mitogen-activated protein kinases influence the ability of certain malignant lymphoid cells to undergo apoptosis when treated with glucocorticoid. Activated/phosphorylated JNK and ERK appear to counteract corticoid-dependent apoptosis. Inhibiting these MAPKs restores corticoid sensitivity to a resistant clone of CEM cells. Forskolin, which activates the cAMP pathway, and rapamycin, which inhibits mTOR, also inhibit JNK. Further, the sensitizing treatments result in a largely dexamethasone-dependent increase in the total pool of glucocorticoid receptor phosphorylated at serine 211. The phospho-serine 211 receptor is known to be more potent in activating gene transcription and apoptosis. The interactive effects demonstrated here in reverting resistant cells to corticoid sensitivity could provide therapeutic clinical potential in the treatment of lymphoid malignancies.

## Background

Recent discoveries have shed light on the mechanism by which glucocorticoids (GCs) cause apoptosis of malignant lymphoid cells. The classical context of glucocorticoid receptor (GR) action dictates that upon ligand binding GC, the GR sheds its cytosolic chaperones, translocates to the nucleus, and binds to DNA glucocorticoid response elements (GREs). There, recruitment of appropriate accessory proteins leads to induction or repression of target genes. The GR also can alter gene expression through interactions with heterologous transcription factors. In recent years, it has become clear that these GR activities are strongly affected by "crosstalk" with several major protein kinase signaling pathways. These receive signals from extracellular ligands through their cognate receptors in the plasma membrane and are affected by the redox state of the cell [1-10]. An intricate set of linked mechanisms modulate GC/GR function and help explain how GCs differentially affect various cellular processes within the body. Cell- or tissue-specific differences in the strength and composition of such crosstalk pathways may explain how some lymphoid cells with functional GRs escape apoptosis despite pharmacological treatment with GCs. By use of clones from the CEM line of childhood acute lymphoblastic leukemia (ALL) cells, we have shown that the cAMP/protein kinase A (PKA) and mitogen- activated protein kinase (MAPK) signaling pathways strongly influence the response of human ALL cells to GC. These findings have recently been confirmed [11]. Activation of PKA by use of forskolin (FSK) to elevate cell cAMP levels synergizes with GC to kill inherently GC-sensitive CEM clones. More strikingly, FSK can render an inherently GC-resistant CEM clone fully sensitive to GC-evoked apoptosis [9]. This result was confirmed and extended by others, who used a different CEM clone, CEM GH, to show that blocking cAMP phosphodiesterase activity enhanced sensitivity to GC [12]. Though blocking the type-4 phosphodiesterase PDE4 did not potentiate GC's in the uncloned CCRF CEM line, treatment with FSK did. The same group found that blocking PDE4 in B-cell chronic lymphocytic leukemia was effective in enhancing GC apoptotic action. There clearly is a connection between the PKA and GC pathways, though exactly which PKA substrates account for the enhancement of GC apoptotic activity in lymphoid cells remains to be clarified.

The MAPKs are a second important interactive pathway that affects the GR. A tiered system of protein kinases leads from cell surface receptors to the three major classes of MAPKs: extra-cellular signal-regulated kinase (ERK), c-Jun N-terminal kinase (JNK), and p38, each of which contains several isoforms [10,13,14]. Considerable pathway redundancy and overlap exists prior to the MAP kinase kinases (MKKs), but at MKKs relative specificity of substrates occurs, as the activated MKKs phosphorylate and activate particular MAPKs. Upon phosphorylation MAPK enzymatic activity increases as much as 1,000-fold to phosphorylate in turn their respective sets of target proteins, culminating in a biological response [14]. MAPKs are subsequently inactivated through the action of a family of dual-specificity protein phosphatases. Crosstalk between the GC and MAPK pathways has been under recent scrutiny, and several studies including our own demonstrate a direct role for MAPK regulation of GR action, including effects on the chemotherapeutic sensitivity of leukemic cells [1,15-17]. Our previous results in clones of GC-sensitive ALL cells showed that ERK and JNK protected against GC-dependent apoptosis, whereas p38 activation promoted such apoptosis [16]. We showed that p38 could specifically phosphorylate serine 211 (Ser 211) of the GR, resulting in enhanced transcriptional and apoptotic activity.

Herein, we focus on the GC-resistant ALL clone CEM-C1-15, comparing the effects of manipulating the PKA, mTOR, and MAPK pathways on GC sensitivity. All approaches converged on the MAPKs and GR. Direct inhibition of JNK and ERK, for example, allowed CEM-C1-15 cells to be killed by the synthetic GC, dexamethasone (Dex). As it did in the parental clone CEM-C1, treatment with FSK restored GC sensitivity to CEM-C1-15 cells and reduced JNK activity. Recently, the combination of GC with the immunosuppressant rapamycin has shown an ability to restore apoptotic sensitivity to CEM-c1 cells, a camptothecin-resistant CEM clone [18]. We show that rapamycin also diminished JNK activity. Thus, each GC-sensitizing treatment led to an alteration in the cellular balance of ERK and JNK vs. p38 MAPK activity. Furthermore, all the GC-sensitizing treatments resulted in site-specific phosphorylation of the GR at Ser 211 accompanied with an increase in total GR protein. These data support our hypothesis that in certain lymphoid malignancies, the balance between the anti-apoptotic activities of ERK and JNK on the one hand, and the pro-apoptotic activity of p38 on the other, are strong determinants of the cellular response to GC.

## Results

### MAPK protein levels remain unchanged after Dex treatment in CEM cells

**Figure 1:**
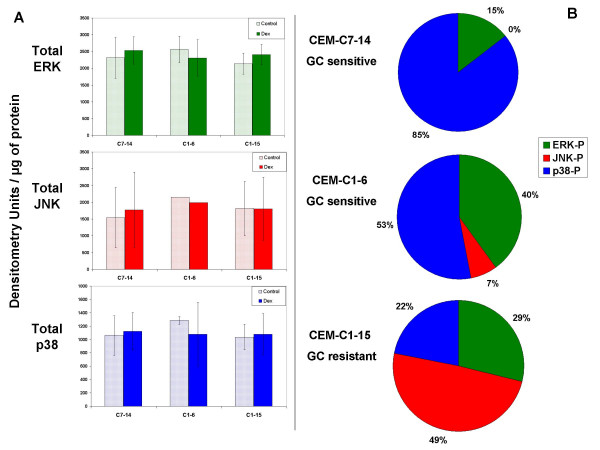
**MAPK protein levels and proportions of phosphorylated MAPKs in GC-sensitive and resistant CEM clones**. (A) Immunoblot of total protein of each MAPK class showed no significant differences in quantity within each class between clones or after Dex treatment, n = 4 for JNK and p38, 5 for ERK. (B) Percentages of phosphorylated MAPKs (ERK, JNK, and p38) in the presence of Dex per μg protein. (see Methods)

Preliminary experiments established the linear range of the immunochemical reactions for ERK, JNK, and p38. Working within this range, total and phosphorylated ERK, JNK, and p38 were estimated quantitatively by image analysis. In four, or for ERK five, independent experiments, none of the MAPK's showed variation in the basal state or after Dex treatment (Fig. 1A). Therefore, the amount of each immunochemically detected MAPK could be expressed in terms of total extract protein. This allowed normalization of the data for phosphorylated MAPKs over several experiments.

### MAPK phosphorylation states in Dex-sensitive vs. Dex-resistant CEM clones

**Figure 2:**
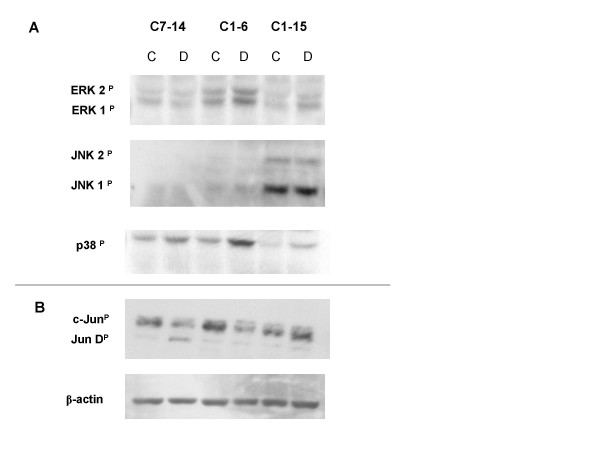
**Effect of Dex on the phosphorylation state of major MAPKs and the JNK substrate c-Jun in basal and Dex treated sensitive and resistant CEM clones**. (A) Cell clones CEM-C7-14 and CEM-C1-6 (both Dex-sensitive) as well as CEM-C1-15 (Dex-resistant) were treated with ethanol vehicle (C) or 1 μM Dex (D) for 20–24 hours. Cell lysates were evaluated by use of phospho-specific antibodies. Shown is a typical immunoblot for phosphorylated MAPK, from 4–5 independent experiments. (B) One of three immunoblots for phosphorylated c-Jun.

Figure 2A depicts the immunochemical reactions for phosphorylated MAPKs in GC-sensitive CEM-C7-14 and CEM-C1-6 cells alongside the GC-resistant CEM-C1-15 clone before and after 20–24 hour incubation in 1 μM Dex. After that interval, the sensitive cells are poised to begin recruitment into apoptosis [19,20]. Our earlier results showed that phosphorylated ERK and JNK protected against GC-evoked apoptosis in sensitive clones, whereas p38 MAPK enhanced it [16]. We therefore examined phosphorylated MAPK levels in the resistant clone CEM-C1-15. The pattern of basal levels of phosphorylated/activated MAPKs are clearly different in clone C1-15 compared to the sensitive clones. The data also show a striking elevation of phosphorylated JNK in C1-15 cells compared to either sensitive clone; Dex treatment did not affect the quantity of phosphorylated JNK in any clone. CEM-C1-6 and CEM-C7-14 both had greatly reduced basal JNK phosphorylation relative to CEM-C1-15. JNK phosphorylation generally is believed to correspond to activation; however, to confirm differential JNK activity, we assayed cell extracts for their ability to phosphorylate c-Jun, a JNK substrate. With Dex treatment, c-Jun phosphorylation was reduced in the sensitive clones, whereas it was increased in C1-15 cells (Fig. 2B). By this index, the cellular differential in JNK activity between sensitive and resistant seen in the basal state actually increases after Dex exposure. The results in Fig. 2A and 2B are consistent with the hypothesis that JNK has a protective effect against Dex-dependent apoptosis.

Furthermore, our earliest data demonstrated that in the sensitive clones Dex-dependent p38 phosphorylation/activation is pro-apoptotic [16]. Analysis of p38 phosphorylation showed higher basal levels in both sensitive clones relative to clone C1-15 (Fig. 2A). The level of p38 phosphorylation increased in response to Dex treatment in all three cell clones, but the weakest increase was seen in CEM-C1-15, in which the maximum level reached after Dex treatment was below the basal amounts in the sensitive clones (Fig. 2A). Basal levels of phosphorylated ERK (anti-apoptotic) were highest in C1-6 cells, intermediate in C7-14 cells, and lowest in the resistant C1-15 cells. There appeared to be an increase of phosphorylated ERK in response to Dex treatment in C1 subclones C1-6 and C1-15, not in C7-14 cells. Blocking the anti-apoptotic activity of both ERK and JNK in the sensitive clones maximized, and inhibition of p38 reduced, Dex-dependent apoptosis [16]. The results shown in Fig. 2 support the hypothesis that the balance between the combined anti-apoptotic activities of ERK + JNK and the pro-apoptotic activity of p38 is a strong determinant of the cellular apoptotic response to Dex. Fig. 1B shows the proportions of phospho-p38 (blue), -ERK (green) and -JNK (red) after Dex treatment in the three clones. It is obvious that the relative amount of phospho-(ERK + JNK) is much greater in the resistant clone. Per unit protein, phospho-p38 represents the majority of the phospho-MAPKs in the sensitive clones, but only 22% in the resistant clone. Again, these data are consistent with our hypothesis. A prediction of this hypothesis is that altering the balance of active MAPKs should affect the Dex-dependent apoptosis of CEM-C1-15 cells. We next tested this prediction.

### Inhibition of ERK and JNK activity confers a Dex-sensitive phenotype on GC-resistant CEM-C1-15 cells

**Figure 3:**
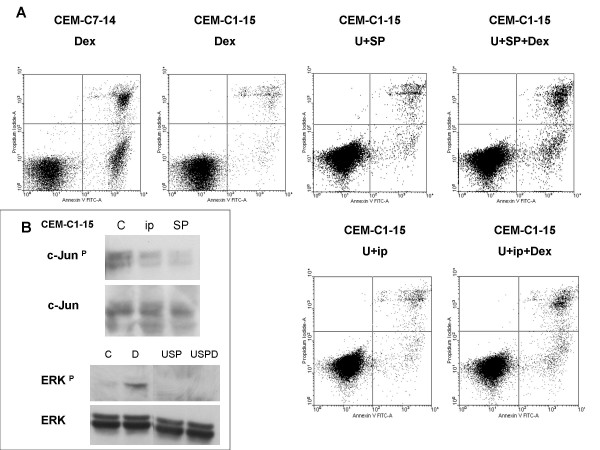
**Inhibition of ERK plus JNK sensitizes CEM-C1-15 cells to Dex-dependent apoptosis**. Flow cytometric analysis of 20,000 collected events for each experiment. (A) Evaluation by Annexin-V staining and PI exclusion assay. CEM-C1-15 cells were pretreated for 24 hours with U0126 (U) plus SP600125 (SP) or cell permeable JNK inhibitory peptide (ip). Dex was then added and after a further 24 hours (for treatments with U+SP or U+SP+Dex) or 72 hours (Dex or U+ip or U+ip+Dex), cells were stained with Annexin-V-FITC/PI and examined by flow cytometry. Cells of sensitive clone CEM-C7-14 treated with Dex only are shown as a positive control. Abscissa: histogram of cells positive for Annexin-V; ordinate: cells positive for PI uptake. Note log scales, lower left quadrant shows viable cells; lower right, Annexin-V positive cells (early apoptosis); upper right, cell positive for both Annexin-V and PI (late apoptosis). Inset B is an assay for the block of ERK and JNK activity. Extracts of CEM-C1-15 cells treated with vehicle (C), ip, or SP were immunochemically tested for phosphorylation of c-Jun, n = 2. Extracts tested for ERK phosphorylation were treated with vehicle (C), Dex (D), U0126 plus SP600125 (USP), or the combination (USPD), n = 1.

**Figure 4:**
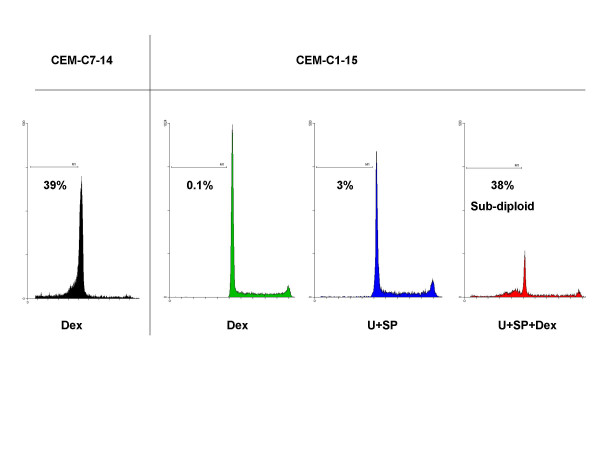
**Sub-diploid DNA content increases upon restoration of Dex-sensitivity through inhibition of ERK and JNK in CEM-C1-15 cells. **CEM-C1-15 cells were treated simultaneously with Dex ± U+SP. Dex-treated CEM-C7-14 cells serve as a positive control for apoptotic response. After 72 hours, nuclear suspensions were evaluated for distribution of DNA content by PI staining. Histograms of cellular DNA content as well as percentages of sub-diploid DNA are presented, example of n = 4.

To further evaluate the roles that ERK and/or JNK play in the resistance of CEM-C1-15 cells to GCs, we pharmacologically blocked ERK activity with U0126 and JNK activity with either the pharmacological JNK-specific inhibitor SP600125 or a cell permeable JNK inhibitory peptide (ip). Inhibition of either ERK or JNK alone partially restored apoptotic sensitivity to Dex in C1-15 cells such that Dex reduced viable cells by 30–40% compared to drug-matched controls (p = 0.03, data not shown). CEM-C1-15 cells undergo apoptosis in response to Dex in the presence of the specific pharmacological inhibitors of ERK plus JNK, whereas ERK plus JNK inhibition alone had very little effect on cell viability, though it greatly slowed cellular growth (data not shown). Fig. 3A shows results using Annexin-V to stain for membrane phosphatidlyserine eversion, a hallmark of early stage apoptosis, combined with propidium iodide (PI) uptake to evaluate cells whose membranes had been compromised. Apoptotic cells appear in the quadrants on the right. Staining with Annexin-V only indicates early or pre-apoptosis (lower right quadrant); staining with both Annexin-V and PI indicate full-blown apoptosis (upper right quadrant). Data from C7-14 cells treated with Dex alone are shown as a positive control. In C7-14 cells, Dex exposure clearly produced apoptosis, but in C1-15 cells, pharmacological inhibitors alone or Dex alone produced little apoptosis. Dex and the inhibitors in combination caused an increase in both early and late apoptotic populations in C1-15 cells. Fig. 3A also depicts an experiment in which JNK was partially inhibited by use of ip. Inhibition of both JNK and ERK again renders the cells susceptible to Dex-evoked apoptosis, but the peptide was not as effective an inhibitor of JNK activity as SP600125. Fig. 3B shows that ip is less effective at reducing phospho-c-Jun than SP and produces a corresponding lesser sensitization to Dex. Fig. 3B also demonstrates the ability of the MAPK drug combination to inhibit phospho-ERK. Fig. 4 shows flow cytometry histograms of the DNA in permeabilized cells stained with PI. The sub-diploid (apoptotic) fraction of CEM-C1-15 cells to the left of the G1 peak was increased by the combination of both inhibitors plus Dex to an extent similar to that of the sensitive CEM-C7-14 cells exposed to Dex alone. Such data shows the state of cells with membranes still intact, but not the lethal effect of the treatment, which can only be determined by quantifying viable cell numbers as well.

**Figure 5:**
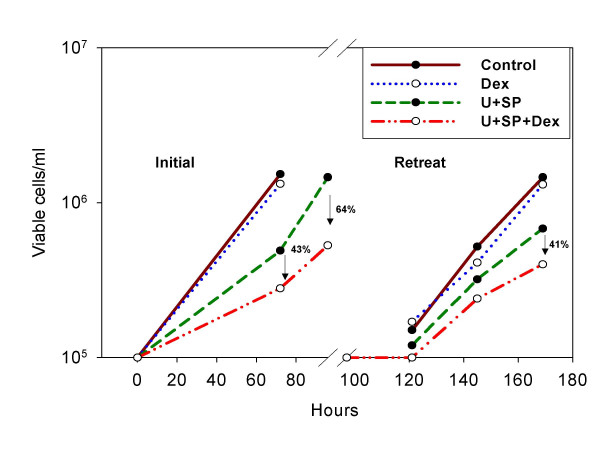
**CEM-C1-15 cells that survive treatment with MAPK inhibitors plus Dex remain sensitive upon retreatment**. CEM-C1-15 cells were treated with vehicle (Control), Dex, U0126 (U) and SP600125 (SP) in the combinations shown (Initial). After 96 hours, the drugs were diluted by centrifugation, washed, and the cells immediately reincubated in fresh growth medium. The drug combinations were then reintroduced (Retreat). At the time points shown, viable cells were counted by trypan blue dye exclusion assay, n = 2. Ordinate, note log scale.

Since the restored sensitivity of CEM-C1-15 to Dex did not result in a complete loss of viable cells in a single round of treatment, we evaluated the sensitivity of the residual cell population. These cells could represent a completely resistant subpopulation or a population that survived due to several factors, e.g. inadequate block of ERK and JNK or metabolism of the blocking drugs. Cells that survived the initial treatment of double MAPK block plus Dex were washed, grown for 24 hours in the absence of the drugs and immediately retreated. The outgrown cell population responded similarly as did naïve cells (Fig. 5). In two experiments, this cycle could be serially repeated four times, after which the experiments were halted. We concluded that the incomplete cell kill by the combination of Dex and MAPK blocking drugs was not due to selection of a permanently resistant subpopulation.

### FSK treatment of CEM-C1-15 cells sensitizes to Dex and suppresses phosphorylated JNK

We have shown earlier that FSK alone slowed growth of CEM-C1 cell numbers with little kill and that addition of Dex caused apoptosis [9]. The same effects were seen in C1 subclone 15 (Table 1). The table shows that FSK reduced cell numbers, but caused little change in viability. Adding Dex dramatically lowered total viable cells.

**Table 1:** Forskolin and Rapamycin sensitize CEM-C1-15 cells to Dex.

Dex resistant CEM-C1-15	C	Dex	F	F+Dex	R	R+Dex
Viable Cells/ml (× 10e5)	19	16	7.1	1.4	3.6	1.7
Significance (VC/ml, C vs Rx) p =		0.11		0.01		0.004
% of Drug matched control		84		20		47
% Culture viability	99	98	90	38	97	87
Dex sensitive CEM-C7-14						
Viable Cells/ml (× 10e5)	14	3.2				
Significance (VC/ml, C vs Rx) p =		0.002				
% of Drug matched control		23				
% Culture viability	98	54				

Cells were plated in triplicate wells at 1 × 10^5 ^viable cells/ml and treated with FSK or rapamycin (C = vehicle, F =FSK, R = rapamycin). Dex was added within 5 minutes of FSK addition and 6 hours after addition of rapamycin. Cells were evaluated by trypan blue exclusion at 48 hours for FSK and 72 hours for rapamycin and Dex alone treatments; p-value indicates results of a paired t-test on viable cell numbers for each drug matched sample. To evaluate the Dex effect for each treatment, viable cell numbers for each drug and drug plus Dex were compared and expressed as % of drug matched control. Percent culture viability indicates the percentage of cells able to exclude trypan blue dye (viable cells) at the time of evaluation. Dex-sensitive CEM-C7-14 is included as a positive control for Dex sensitivity, n = 1–3.

**Figure 6:**
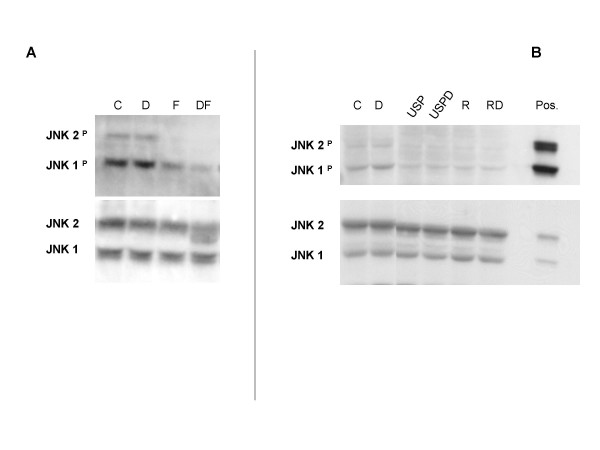
**Inhibition of JNK phosphorylation correlates with treatments that restore Dex-sensitivity to CEM-C1-15 cells**. (A) CEM-C1-15 cells were at once treated with vehicle control (C), Dex (D), FSK (F), or the combination of FSK plus Dex (DF) for 24 hours. JNK was evaluated by immunoblot for phosphorylation of Threonine^183^/Tyrosine^185 ^and total protein, example of n = 4. (B) CEM-C1-15 cells were pretreated with vehicle control (C), U0126 (U) plus SP600125 (SP), rapamycin (R), or combinations of these drugs for 6 hours before adding Dex (D) for an additional 18 hours. JNK was evaluated as in panel A, example of n = 2–4 for various combinations of treatments.

To determine whether there was a convergence of pathways, we examined the state of JNK and ERK phosphorylation in these cells. FSK with or without Dex lowered phospho-JNK (Fig. 6A), but had little effect on phospho-ERK when compared to Dex alone (data not shown). FSK alone had little effect on total JNK protein (6A, lower panel), but the addition of Dex caused an alteration in the migration of the JNK2 isoform. Further investigation of this phenomenon will be required to identify the nature of the new, faster-running band.

### Rapamycin lowers phosphorylated JNK and converts CEM-C1-15 cells to a Dex-sensitive state

Rapamycin is an immunosuppressive drug known to interact with the mammalian target mTOR and thereby to inhibit cap-dependent mRNA translation [21]. Such drugs show promise for treatment of cancers. It has been shown recently that treatment of CEM-c1 cells with rapamycin renders them sensitive to Dex-dependent apoptosis [18]. We tested for the effect of rapamycin on the MAPK system and found that rapamycin lowers phosphorylated JNK levels (Fig. 6B) and renders CEM-C1-15 cells sensitive to Dex-evoked apoptosis (Table 1). Rapamycin plus Dex treatment did not diminish phospho-ERK levels compared to Dex treatment alone (data not shown). The data from Fig. 6B show that rapamycin reduces the phosphorylation of JNKs 1 and 2 to an extent similar to that caused by combination of the MAPK inhibitors U0126 and SP600125.

### Site-specific phosphorylation and auto-induction of the GR correlates with sensitization of CEM-C1-15 cells to Dex-dependent apoptosis

**Figure 7:**
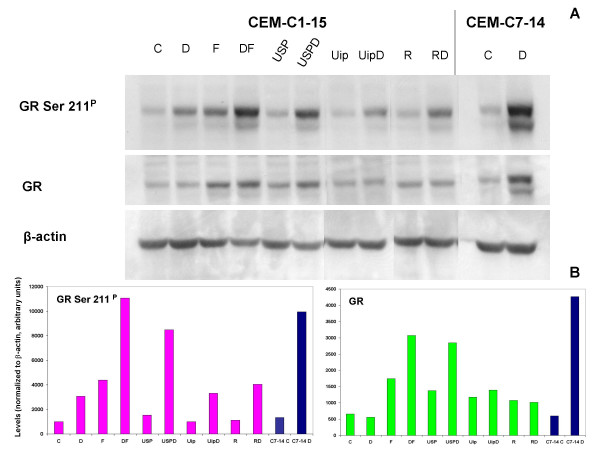
**Evaluation of GR protein and phosphorylation state in CEM-C1-15 cells rendered sensitive to Dex by several treatments**. (A)CEM-C1-15 cells were pretreated with vehicle control (C), FSK (F), U0126 (U) plus SP600125 (SP), U0126 plus (ip), rapamycin (R), or combinations of these treatments for 5.5 hours before adding Dex (D) for an additional 17 hours. Dex-sensitive CEM-C7-14 cells were treated with vehicle control or Dex for 17 hours, and results are included as a positive control. Equal amounts of protein were added to each lane. The results were evaluated by immunoblot with antibodies against phospho-GR (Ser 211), total GR, and β-actin, n = 2–4 for various combinations of treatments. (B) Densitometric analysis of immunoblot from (A). All bands were normalized to β-actin; bars = arbitrary units of variable/densitometry units of β-actin for each corresponding lane.

In Dex-sensitive CEM clones, treatment with Dex results in phosphorylation of the GR at Ser 211, an effect important for enhanced transcriptional and apoptotic potency of the GR and in part dependent on p38 MAPK [16]. It has been shown by others that the Dex-dependent auto-induction of GR correlates with later apoptosis in these cells [22]. Consequently we evaluated the status of the GR in CEM-C1-15 cells after various treatments by using immunoblotting for phopho-Ser 211 GR and total GR with subsequent densitometry analysis (Fig. 7A and 7B). GR protein increased after treatment with FSK and more so after FSK plus Dex, by pharmacologic inhibitors of JNK and ERK alone and also plus Dex, by rapamycin alone, without further increase when Dex was added, by Uip, and similarly by Uip plus Dex. (Fig. 7B, GR).

Immunoblotting with antibodies specific for GR phospho-Ser 211 indicated a weak increase in phosphorylation state with no increase in GR protein in response to Dex treatment alone in CEM-C1-15 cells (Fig. 7A CEM-C1-15 and B, lanes/bars C and D) compared to the effect of Dex in the sensitive CEM-C7-14 clone (Fig. 7A and 7B, CEM-C7-14, lanes/bars C and D). The Dex-dependent phosphorylation of GR Ser 211 in C1-15 cells was distinctly enhanced when JNK and ERK were blocked pharmacologically. The inhibitors alone had minimal effect on basal low levels of GR phospho-Ser 211 (Fig. 7A and 7B, compare lanes/bars C, USP, and USPD). Use of the weaker peptide inhibitor ip to inhibit JNK produced qualitatively similar but lesser changes. Treatment with rapamycin, while increasing GR protein, did not increase its phosphorylation at Ser 211, but Ser 211 did increase with the addition of Dex.

FSK alone increased GR protein and GR Ser 211 phosphorylation; Dex enhanced both effects. The net effect of Dex in combination with the various drugs resulted in every case in more total phospho-Ser 211 GR in the Dex-treated sensitized cells.

**Figure 8:**
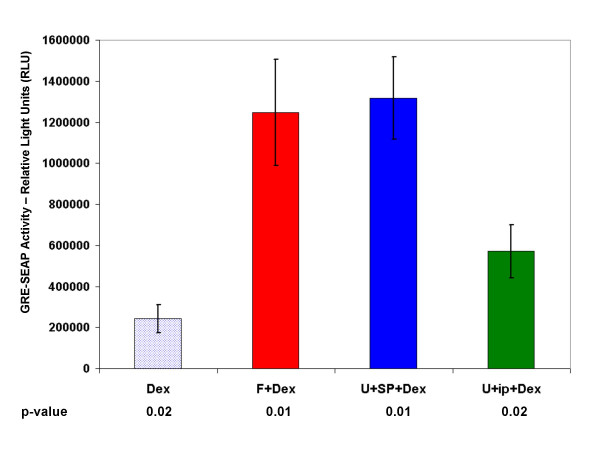
**Transcriptional potency of the GR is enhanced upon restoration of Dex-sensitivity to CEM-C1-15 cells**. CEM-C1-15 cells were electroporated with a plasmid expressing three GREs in tandem fused to a SEAP reporter. Cells were subsequently pretreated with vehicle control, FSK (F), U0126 (U) plus SP600125 (SP), U0126 plus (ip), or combinations of these treatments for 6 hours before adding Dex for an additional 24 hours with subsequent evaluation of SEAP activity. Drug treatment alone was subtracted as background and shown is the Dex effect on each treatment. Error bars: 1 standard deviation, average of 3 independent replicates; p-value is based on a t-test for matched drug treatments ± Dex, n = 2–3 for various combinations of treatments.

We evaluated intracellular transcriptional activity of the GR by use of a transfected promoter-reporter plasmid encoding GREs fused to a secreted alkaline phosphatase (SEAP) reporter. Upon treatment with U0126 and SP600125 in combination with Dex, transcriptional activity of the GR was significantly increased over treatment with Dex alone (Fig. 8). Substitution of the ip to JNK still supported increased GR transcriptional activity, but to a lesser extent; again consistent with the fact that the peptide fails to fully inhibit JNK. Thus, inhibition of JNK and ERK, which renders otherwise resistant C1-15 cells sensitive to Dex-dependent apoptosis, also supported Dex-dependent increases in GR phosphorylation at Ser 211, total GR protein, and the activity of the GR. The combination of FSK and Dex resulted in as great an increase in SEAP induction as did blocking ERK and JNK in combination with Dex. Cotreatment with rapamycin plus Dex, however, though enhancing apoptosis, decreased steroid-dependent induction of SEAP activity from the GRE-SEAP construct (data not shown). This is no doubt due to inhibition of SEAP mRNA translation by rapamycin; the drug does not inhibit induction of reporter mRNA [23].

## Discussion

In the search for the GC-driven pathway to malignant lymphoid cell apoptosis, clones from the CEM line of ALL cells have proven extremely useful. We compared basal and Dex-induced levels of genes in three closely related clones: one inherently sensitive to Dex-induced apoptosis; a sister clone that is inherently resistant, and a third revertant to sensitive from their resistant parental clone [19]. Earlier data revealed that activation of the MAPK p38 contributed to the apoptotic outcome, whereas MAPKs, JNK and ERK acted to prevent or ameliorate Dex-dependent apoptosis [16]. Consistent with this finding, we show that basal levels of phosphorylated JNK were strikingly elevated in the resistant CEM-C1-15 clone compared to the sensitive clones. In addition, phospho-ERK was increased by Dex in these cells. Its sister clone CEM-C1-6, a revertant to sensitive, had greatly lowered phospho-JNK although phospho-ERK remained the highest of the three tested CEM clones. This suggested that combined contributions from JNK and ERK favored Dex resistance. The anti-apoptotic effect of ERK in relation to GCs in a different clone of CEM cells has recently been reported [11]. We hypothesized that the elevated levels of phospho-(JNK + ERK) were at least partly responsible for the resistance to Dex of CEM-C1-15 cells. We tested that hypothesis by blocking JNK and ERK activity in clone C1-15. Single blocks produced partial Dex sensitivity, but a better conversion to the Dex-sensitive phenotype was achieved by blocking both JNK and ERK. We noted that Dex did not directly cause changes in the protein levels of the MAPKs; yet in lymphoid cells, altered gene transcription is required for corticoid-dependent apoptosis. Thus, post-translational alterations in phosphorylation/activation of the MAPKs depend on Dex-dependent changes in transcription of genes other than those of the MAPKs themselves.

Recent time-course analysis of Dex-dependent changes in mRNA levels in two Dex-sensitive clones suggested that a network of induced and repressed genes coordinately affect MAPK phosphorylation (submitted). We also observed a marked difference in the c-Jun phosphorylation pattern in response to Dex between the sensitive and resistant subclones. CEM-C7-14 had undetectable and CEM-C1-6 had greatly reduced phospho-JNK levels while still showing c-Jun phosphorylation. It has been previously reported that c-Jun can be phosphorylated by several other protein kinases besides JNK [24]. We suggest that the actions of other kinases and/or phosphatases influenced by Dex in the sensitive CEM clones are intervening at the level of c-Jun phosphorylation. Though JNK and ERK both must be blocked to maximize the conversion of CEM-C1-15 cells to Dex sensitivity, of the two, JNK seemed the more important. This is consistent with the numerous recent reports that emphasize the reliance of several types of malignant lymphoid cells on JNK and/or ERK for viability [25-31].

We had previously observed that activation of the PKA system by use of FSK transformed CEM-C1 cells – the initial resistant clone – into cells Dex-sensitive for apoptosis [9]. After confirming that the same held true for subclone C1-15, we tested for a point of convergence at JNK. The data show that treatment with FSK lowers the levels of phosphorylated JNK and this effect may be enhanced by the addition of Dex. FSK is known to activate phosphatases that dephosphorylate MAPKs [32-34]. Since FSK alone lowers phospho-JNK and inhibits growth, but does not kill either Dex-sensitive or Dex-resistant cells, the lowered phospho-JNK must provide a background state in which Dex can produce additional alterations necessary to initiate cell death. FSK treatment consistently renders C1-15 cells more sensitive to Dex than other treatments that reduce phospho-JNK equally well. Thus, activation of PKA contributes additional factors to Dex-dependent cell death.

That JNK is a nexus for pathway interactions in Dex-dependent apoptosis is further suggested by use of rapamycin. This drug blocks cap-dependent mRNA translation through the mTOR pathway [21,23] and recently has been shown to render CEM-c1 cells partially sensitive to Dex [18]. We confirmed this result in CEM-C1-15 and now find that rapamycin inhibits JNK phosphorylation in the CEM system. Rapamycin inhibits translation of some, not all mRNAs, and it has been suggested that Dex induction of those not blocked by rapamycin is the key to apoptosis [23]. Our results suggest that reduction of the levels of anti-apoptotic phospho-JNK is a component of the sensitization to Dex caused by rapamycin. Undoubtedly, other rapamycin effects are involved as well.

**Figure 9:**
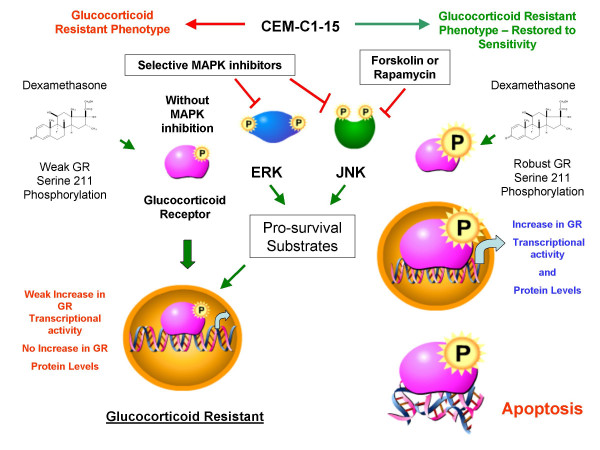
**Agents that restore GC-sensitivity to CEM-C1-15 cells converge on the GR and MAPK pathways**. Dex-resistant CEM-C1-15 cells in their natural state harbor high levels of pro-survival, anti-apoptotic active JNK (green symbol) and low levels of active ERK which is Dex-inducible (blue symbol). The cells also contain GR (pink symbol). The sequence on the left side shows the result in CEM cells which resist Dex-dependent apoptosis. In this case, added Dex mediates a weak increase in GR phospho-Ser 211 as well as GRE reporter driven activity, but no increase in GR protein levels, and the cells remain resistant. The sequence on the right depicts the results when CEM-C1-15 cells are treated with combinations of Dex and MAPK inhibitors, FSK, or rapamycin. These treated cells convert to a GC-sensitive phenotype. All treatments converge at inhibition of the JNK MAPK pathway. Upon restoration of the Dex-sensitive phenotype, a robust increase in GR phospho-Ser 211, GR protein, and transcriptional activity is observed. These effects culminate in an apoptotic response.

The GR is a second point of convergence between the drugs employed herein. Phosphorylation of Ser 211 in the human GR increases the transcriptional and apoptotic potency of the receptor, and mutation of Ser 211 to a non-phosphorylatable amino acid reduces GR-based apoptosis and gene transcription [16]. The p38 MAPK is capable of carrying out this phosphorylation, and other kinases may do so as well [16,35]. All of the sensitizing treatments lead to an increase in GR protein and in some cases adding Dex further increases GR. When Dex is added, the proportion of phospho-Ser 211 GR is increased. The result is that the total amount of phospho-Ser 211 is increased in cells that the drugs have rendered sensitive to Dex. This condition therefore correlates with diminished JNK activity and increased GR apoptotic/transcriptional potency. A scheme outlining these effects is presented in Fig. 9.

## Conclusion

GCs appear to have a generalized metabolic effect upon most if not all of the cells in the human body. Synthetic GCs, such as Dex, in pharmacological doses have been used successfully in the treatment of lymphoid malignancies for many years. However, not all lymphoid cells are responsive to GCs. While it is well documented that GCs act through the GR to produce its multitude of responses, the complete pathway by which response or no response is accomplished is not clearly understood. We present in this paper information clarifying the relationship between the GR and the MAPK pathways in lymphoid cells. In Dex-sensitive cells the intracellular balance of MAPKs ERK and JNK which are anti-apoptotic in their active phosphorylated form must be maintained in a reduced state while the MAPK p38 which is pro-apoptotic must be enhanced. GC-resistant lymphoid cells may be rendered sensitive by: (1) inhibition of JNK and ERK activity, (2) stimulation of the cAMP/PKA pathway with FSK, or (3) inhibition of mTOR with rapamycin. All three treatments in  combination with Dex alter the balance of cellular phospho-MAPKs by lowering JNK phosphorylation, stimulating site-specific, activity-enhancing phosphorylation of the GR at Ser 211, and increasing total GR protein levels culminating in an apoptotic response. Thus JNK serves as the convergence point between the GR and MAPK pathways. We propose that the interactive effects found in CEM cells may apply to other hematological malignancies as well. Tests of this hypothesis are underway in our laboratory.

## Methods

### Cell culture and drug concentrations

The CCRF-CEM human cell line was isolated from a patient with ALL [36]. Early Dex-sensitive (C7) and Dex-resistant (C1) clones were subcloned to give CEM-C7-14, sensitive to Dex-evoked apoptosis; CEM-C1-6, a Dex-sensitive spontaneous revertant; and Dex-resistant CEM-C1-15 [19]. Cells were grown in RPMI 1640 (Cellgro Media Tech, Herndon, VA) pH 7.4, supplemented with 5% fetal bovine serum (FBS) (Atlanta Biologicals, Norcross, GA). Cells were cultured at 37°C in a humidified atmosphere of 95% air, 5% CO_2 _and subcultured regularly to ensure logarithmic growth. Cell viability was determined by trypan blue dye (Sigma-Aldrich, St. Louis, MO) exclusion using a manual hemacytometer or Vi-cell automated cell (Beckman Coulter, Miami, FL) counting. Cells were treated as appropriate for each experiment with vehicle (ethanol/DMSO/and/or sterile HPLC-grade water < 0.1% final concentration), 1 μM Dex, 4.5 μM JNK inhibitor (SP600125), 2.6 μM MEK inhibitor (U0126), 10 μM FSK, 1 μM ip, 10 nM rapamycin, or various combinations of each. All chemicals were from Sigma-Aldrich, Burdick and Jackson, Muskegon, MI or Calbiochem, San Diego, CA. For all experiments (n) equals the range of subsets of independent experiments.

### Immunochemical analysis

Cells in mid-logarithmic growth were treated with the various drugs as appropriate for each experiment. To ensure accurate protein identification, positive controls were generated by known strong inducers of MAPKs. We treated the cells with 50 ng/ml phorbol 12-myristate 13-acetate plus 1 μg/ml phytohemagglutinin for phospho-ERK, ultraviolet light for phospho-c-Jun (Cell Signaling Technology, Beverly MA), and 100 nM anisomycin for phospho-JNK and p38. These controls were run in independent lanes on each blot. After the various experimental or positive control treatments, cells were harvested at various time-points thereafter by centrifuging at 1,000 rpm for 5 minutes in a Beckman Allegra 6R centrifuge at 22°C. The cells were resuspended in 10 ml/22°C phosphate buffered isotonic saline pH 7.4 (PBS), and recentrifuged. The washed cells were transferred to 1.5 ml centrifuge tubes and lysed on ice using 4°C M-per cell lysis buffer (Pierce, Rockford, IL) supplemented with 1 × general protease inhibitor cocktail, 10 mM sodium fluoride, and 1 mM sodium orthovanadate (Sigma-Aldrich). Cellular debris was pelleted at 13,000 rpm for 10 minutes at 4°C in a Beckman microfuge. The clarified samples were transferred to fresh, pre-chilled microfuge tubes, and the protein concentration was estimated using BCA (Pierce). The lysate was mixed with 5 × SDS-PAGE sample buffer supplemented with 2% 2-mercaptoethanol and heated to 100°C for 5 minutes. Equally loaded proteins were separated by electrophoresis on 8% SDS-PAGE gels and transferred to a PVDF membrane (Bio-Rad, Hercules, CA or Amersham Pharmacia Biotech, Piscataway, NJ) using a semi-dry electroblotter (Hoefer, San Francisco, CA). Membranes were washed with Tris-buffered saline/Tween-20 (TBST) (140 mM sodium chloride, 20 mM Tris, 0.1% Tween-20, pH 7.6) and blocked for 1 hour in TBST supplemented with 5% non-fat dry milk. Membranes were rewashed and placed in a solution of TBST plus 5% bovine serum albumin containing either an antibody to phospho-(Threonine^202 ^and Tyrosine^204^) -specific to ERK MAPK, or phospho-(Threonine^183 ^and Tyrosine^185^) -specific to JNK MAPK, or phospho-(Threonine^180 ^and Tyrosine^182^) -specific to p38 MAPK (Cell Signaling Technology), or to phospho-c-Jun (Serine^73^) (Calbiochem), or phospho-GR (Serine^211^) (Cell Signaling Technology), or to phosphorylation state independent ERK MAPK (Calbiochem), or JNK MAPK (Cell Signaling Technology), or p38 MAPK (Calbiochem), or GR (Affinity Bioreagents, Golden, CO), or β-actin (Santa Cruz Biotechnology, Santa Cruz, CA) and incubated for 16 hours at 4°C with gentle agitation. Membranes were subsequently washed with TBST and probed with horseradish peroxidase goat/anti-rabbit secondary antibody for 1 hour at 22°C. After rewashing, the membranes were saturated with horseradish peroxide substrate ECL (Amersham Pharmacia Biotech) and exposed to Blue Lite Autorad Film (ISC BioExpress, Kaysville, UT) for various times to ensure linearity. To analyze both phospho- and total proteins on the same filter, after the initial reaction for phosphoprotein, the membranes were stripped of antibody by incubation in Restore™ buffer (Pierce) for 1 hour and reprobed with antibody for the appropriate protein. Densiometric analysis was accomplished using AlphaEase FC™ software (Alpha Innotech Corporation, San Leandro, CA).

### Calculation of proportional MAPK activity

Preliminary experiments established the linear range for directions of immunochemical reaction for ERK, JNK, and p38. Working within this range, total and phosphorylated ERK, JNK, and p38 were estimated quantitatively by image analysis. In 4- or for ERK 5-independent experiments, none of the MAPK's showed variation in the basal state or after Dex treatment. Therefore, the amount of each immunochemically detected MAPK could be expressed in terms of total extract protein (adjusted to X/ml in every experiment). The relative phosphorylated forms of each MAPK, estimated immunochemically, could therefore be calculated:

Phosphorylated MAPK XPhospho (ERK + JNK + p38) (total μg protein)−1=Fraction of X/total phospho MAPK
 MathType@MTEF@5@5@+=feaafiart1ev1aaatCvAUfKttLearuWrP9MDH5MBPbIqV92AaeXatLxBI9gBaebbnrfifHhDYfgasaacH8akY=wiFfYdH8Gipec8Eeeu0xXdbba9frFj0=OqFfea0dXdd9vqai=hGuQ8kuc9pgc9s8qqaq=dirpe0xb9q8qiLsFr0=vr0=vr0dc8meaabaqaciaacaGaaeqabaqabeGadaaakeaafaqaaeGabqaabaGaeeiuaaLaeeiAaGMaee4Ba8Maee4CamNaeeiCaaNaeeiAaGMaee4Ba8MaeeOCaiNaeeyEaKNaeeiBaWMaeeyyaeMaeeiDaqNaeeyzauMaeeizaqMaeeiiaaIaeeyta0KaeeyqaeKaeeiuaaLaee4saSKaeeiiaaIaeeiwaGfabaGaeeiuaaLaeeiAaGMaee4Ba8Maee4CamNaeeiCaaNaeeiAaGMaee4Ba8MaeeiiaaIaeeikaGIaeeyrauKaeeOuaiLaee4saSKaeeiiaacccaGae83kaSIaeeiiaaIaeeOsaOKaeeOta4Kaee4saSKaeeiiaaIae83kaSIaeeiiaaIaeeiCaaNaee4mamJaeeioaGJaeeykaKIaeeiiaaIaeeikaGIaeeiDaqNaee4Ba8MaeeiDaqNaeeyyaeMaeeiBaWMaeeiiaaIaeqiVd0Maee4zaCMaeeiiaaIaeeiCaaNaeeOCaiNaee4Ba8MaeeiDaqNaeeyzauMaeeyAaKMaeeOBa4MaeeykaKYaaWbaaSqabeaacqWFsislcqqGXaqmaaaaaOGaeyypa0JaeeOrayKaeeOCaiNaeeyyaeMaee4yamMaeeiDaqNaeeyAaKMaee4Ba8MaeeOBa4MaeeiiaaIaee4Ba8MaeeOzayMaeeiiaaIaeeiwaGLaee4la8IaeeiDaqNaee4Ba8MaeeiDaqNaeeyyaeMaeeiBaWMaeeiiaaIaeeiCaaNaeeiAaGMaee4Ba8Maee4CamNaeeiCaaNaeeiAaGMaee4Ba8MaeeiiaaIaeeyta0KaeeyqaeKaeeiuaaLaee4saSeaaa@A41C@

### Phosphatidylserine membrane eversion, DNA lysis, and cell cycle evaluation by flow cytometry

Cells were diluted to 5 × 10^4 ^viable cells/ml for CEM-C1-15 and 1 × 10^5 ^viable cells/ml for CEM-C7-14 in 5 ml aliquots in 6-well cell culture dishes. CEM-C1-15 cells were pre-treated with either U0126 plus SP600125 or U0126 plus ip for 24 hours before adding Dex. Cells were harvested at various time-points thereafter by centrifuging at 1,000 rpm for 5 minutes, washed twice with ice-cold PBS, pelleted, and resuspended in 1 × ice-cold binding buffer (10 mM HEPES/NaOH pH 7.4, 140 mM NaCl, 2.5 mM CaCl_2 _from BD Pharmagin (San Jose, CA). 100 μl cell suspension was combined with 5 μl Annexin V-FITC and 10 μl PI (BD Pharmagin) for 15 minutes at 22°C in the dark. 400 μl binding buffer was then added to each sample, and 20,000 cells were processed by flow cytometer (BD FACScanto, BD Biosciences, San Jose, CA) using filters for FITC (488 nm) and PI (> 600 nm).

Cell samples with DNA stained by PI for cell cycle examination were prepared and analyzed as described [16] after treatment for 72 hours with vehicle (ethanol/DMSO), Dex, U0126 plus SP600125, or a combination of the drugs.

### GR activity by GRE-reporter assay

Logarithmically growing CEM-C1-15 cells were collected by centrifugation and washed with 10 ml of sterile 37°C PBS and recollected. The cells were resuspended to a density of 1 × 10^7 ^viable cells/ml in serum-free 37°C RPMI 1640 containing 1.25% DMSO. 400 μl aliquots of the suspension were placed into 0.4 cm gap electroporation cuvettes (Bio-Rad) containing 15 μg of pGRE-SEAP reporter vector (BD Clontech, Mountain View, CA) prepared using a Qiagen maxi-prep kit (Qiagen, Valencia, CA). Cuvettes were electroporated using 975 μF and 270 V with a Gene Pulser II (Bio-Rad). Electroporated cells were diluted in 4 ml (per cuvette) of RPMI 1640 supplemented with 5% FBS and 1.25% DMSO and recultured. Twenty-four hours after electroporation, cells were pelleted, resuspended in fresh RPMI 1640 containing 5% FBS, and viable cells were quantified using trypan blue exclusion. The cultures were diluted to 4 × 10^5 ^viable cells/ml in RPMI 1640 supplemented with 5% FBS, and 500 μl triplicate aliquots per treatment were placed in a 48-well tissue culture plate (Costar, Cambridge, MA). Cells were treated for 24 hours with vehicle (ethanol/DMSO), Dex, U0126, SP600125, ip, rapamycin, or combinations thereof. Samples were subsequently tested for the presence of SEAP using the Great EscAPe SEAP Chemiluminescence Detection Kit (BD Clontech) according to the manufacturer's instructions.

## List of abbreviations

ALL – acute lymphoblastic leukemia

cAMP – cyclic adenosine-3',5'-monophosphate

Dex – dexamethasone

ERK – extra-cellular signal-regulated kinase

FBS – fetal bovine serum

FSK – forskolin

GC – glucocorticoid

GR – glucocorticoid receptor

GRE – glucocorticoid response element

ip – cell permeable JNK inhibitory peptide

JNK – c-Jun N-terminal kinase

MAPK – mitogen-activated protein kinase

MKK – MAP kinase kinase

mTOR – mammalian target of rapamycin

PBS – phosphate-buffered isotonic saline

PKA – protein kinase A

PI – propidium iodide

SEAP – secreted placental alkaline phosphatase

Ser 211 – glucocorticoid receptor serine 211

TBST – Tris-buffered saline containing Tween-20

## Competing interests

The author(s) declare that they have no competing interests.

## Authors' contributions

ALM, ASG, and EBT were involved in experimental design and data analysis. ALM conducted the experiments and wrote the first draft of the manuscript. BHJ and EBT wrote sections and edited the manuscript. EBT served as the principal investigator.
